# One Hundred Glasses a Day

**Published:** 1886-07

**Authors:** 


**One Hundred Glasses a Day.**—"We have a man here who drinks one hundred glasses per day!" This answer was in reply to a question as to how much beer the employees in the Milwaukee breweries consumed. The speaker was a gentleman engaged in the office of an extensive brewing company. "What is the average consumption per man?" "About one and one-fourth casks, or forty glasses daily. As a rule our employees drink fifty one-quarter barrels a day—nearly $100 worth. During warm days this number is increased to sixty quarters and more. Each man generally takes two glasses at one time, which would make the number of visits to the bar about twenty during the day. Allowing three minutes only for each time he knocks off work there is one hour which he loses in this way. Some of the men have to walk a block and further, and it often takes five minutes." "Where is the bar generally located?" "Ours is in a corner of the wash house. We pay a man $60 a month to draw beer for the men, and he earns his money, too. He does nothing else." "Do your employees get as much beer as they can hold since the late strike?" "They receive all they want."

